# Updated Considerations for the Immunopharmacological Aspects of the “Talented mRNA Vaccines”

**DOI:** 10.3390/vaccines11091481

**Published:** 2023-09-12

**Authors:** Cristiana Perrotta, Claudio Fenizia, Carla Carnovale, Marco Pozzi, Daria Trabattoni, Davide Cervia, Emilio Clementi

**Affiliations:** 1 Department of Biomedical and Clinical Sciences (DIBIC), Università degli Studi di Milano, 20157 Milano, Italy; carla.carnovale@unimi.it (C.C.); daria.trabattoni@unimi.it (D.T.); 2 Department of Pathophysiology and Transplantation (DEPT), Università degli Studi di Milano, 20122 Milano, Italy; claudio.fenizia@unimi.it; 3 Scientific Institute IRCCS Eugenio Medea, 23842 Bosisio Parini, Italy; marco.pozzi@lanostrafamiglia.it; 4 Department for Innovation in Biological, Agro-Food and Forest Systems (DIBAF), Università degli Studi della Tuscia, 01100 Viterbo, Italy; d.cervia@unitus.it

**Keywords:** mRNA vaccines, pharmacokinetics, mechanism of action, safety, clinical trials

## Abstract

Messenger RNA (mRNA) vaccines belong to a new class of medications, RNA therapeutics, including both coding and non-coding RNAs. The use of mRNA as a therapy is based on the biological role of mRNA itself, namely its translation into a functional protein. The goal of mRNA vaccines is to produce a specific antigen in cells to elicit an immune response that might be prophylactic or therapeutic. The potential of mRNA as vaccine has been envisaged for years but its efficacy has been clearly demonstrated with the approval of COVID-19 vaccines in 2021. Since then, mRNA vaccines have been in the pipeline for diseases that are still untreatable. There are many advantages of mRNA vaccines over traditional vaccines, including easy and cost-effective production, high safety, and high-level antigen expression. However, the nature of mRNA itself and some technical issues pose challenges associated with the vaccines’ development and use. Here we review the immunological and pharmacological features of mRNA vaccines by discussing their pharmacokinetics, mechanisms of action, and safety, with a particular attention on the advantages and challenges related to their administration. Furthermore, we present an overview of the areas of application and the clinical trials that utilize a mRNA vaccine as a treatment.

## 1. Introduction: An Overview of RNA-Based Drugs

The classical pharmacological approach relies on the role of small molecule drugs (<1000 daltons) to bind active sites of proteins and to alter their function. The ability of a protein to bind small molecules with a high affinity is referred to as druggability. Proteins are druggable in the presence of molecular folds that allows their interaction with other chemical compounds. When these structural features are lacking, proteins might have interesting biological properties but are not good pharmacological targets. Considering that the human genome is likely to have <20,000 protein-coding genes (ca. 1.5% of the whole genome) [1] and that only 600–1500 promising small-molecule drug targets has been estimated within 3000 potentially druggable loci [2,3], the druggable targets for small-molecule therapies appear definitely limited. In addition, small molecules acting as therapeutic agents need a high structural accuracy and thus a complex and challenging developmental/production process. This further reduces the size of the playing field for current small-molecule drug design.

In contrast with small molecule drugs, RNA-based therapeutics consist of different forms of compounds that use an approach not based on protein structure, thus potentially targeting sequences in the whole genome. Among them are synthetic or in vitro produced messenger RNAs (mRNA), which are engineered to mimic endogenous mRNA that encodes for specific peptides or proteins. Exogenous mRNA may be used to replace damaged proteins or present antigens for vaccination [4,5]. Other molecules are the antisense oligonucleotides (ASO), short (ca. 18–30 nucleotides), and synthetic single-stranded nucleic acid polymers: DNA, phosphorothioate DNA, RNA analogs, conformational restricted nucleosides (locked nucleic acids—LNA), or morpholino phosphorodiamidate oligonucleotides complementary to a certain region of RNA. They can be used to modulate gene expression via a range of processes including programmed gene editing, RNA interference, target degradation, non-coding RNA inhibition, gene activation, and altered splicing [6,7]. Synthetic small interfering RNAs (siRNAs) are short (ca. 20–24 nucleotides) non-coding RNA duplexes. The antisense strand is complementary to the coding region of the target mRNA; therefore, siRNA-based drugs enable specific gene-targeted knock-down interacting with the endogenous RNA-induced silencing complex [4,8]. In addition, microRNAs (miRNAs) are a broad class of small, highly conserved non-coding linear RNA molecules that regulate the expression of multiple mRNAs by blocking translation or promoting degradation of the target mRNAs. miRNA-based therapeutics are miRNAs mimics (double-stranded RNA molecules that mimic endogenous miRNAs) and miRNAs inhibitors (single-stranded RNA oligos designed to interfere with endogenous miRNAs) [4,9]. Finally, the group of RNA aptamers includes structured, single-stranded nucleic acid molecules (ca. 20–100 nucleotides) that fold into defined secondary structures and bind to carbohydrates, peptides, proteins, and other molecules. Their tertiary structure and adaptive fit, rather than the sequence, gives high specificity and affinity for the targets. In contrast with other RNA-based therapeutics, aptamers are developed by a combinatorial chemical technology (SELEX—systematic evolution of ligands by exponential enrichment) and not rationally designed. Aptamers act as agonists, antagonists, and even carriers for other drugs. Multi/bispecific aptamers can also be used to concurrently target different markers/pathways [4,10,11].

**Figure 1:**
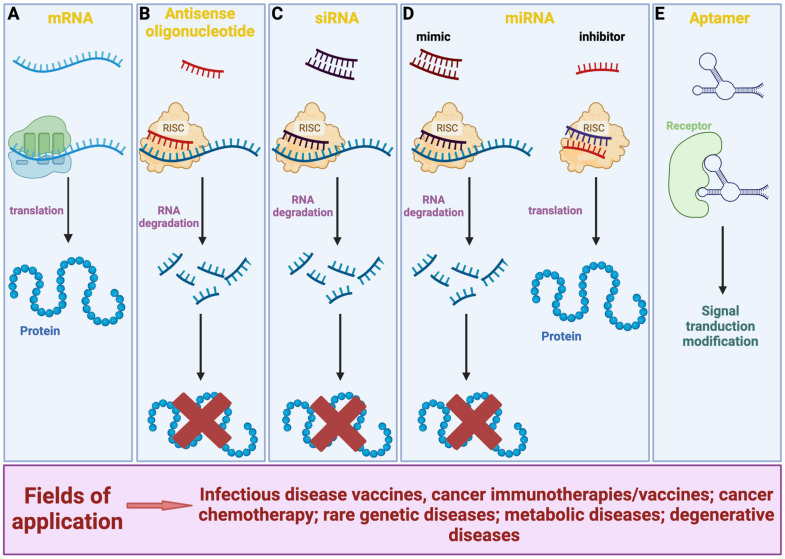
Overview of the various types of RNA-based therapeutics and their modes of action. (**A**) mRNA is translated by ribosomes into proteins. (**B**,**C**) ASO and siRNA are complementary to a target mRNA. They prevent mRNA translation through the association with the RISC complex, thus resulting in its silencing. (**D**) miRNA mimic has a mechanism of action similar to ASO and siRNA; miRNA inhibitor is complementary and binds to an endogenous miRNA thus restoring mRNA translation. (**E**) By binding to a target molecule, aptamer can modulate the signal pathway underlying its action, either positively or negatively. Created with Biorender.com.

Overall, RNA-based therapeutics (Figure 1) are changing the standard of care for many diseases and actualized personalized medicine, representing a rapidly growing area of research and development in the field of pharmacology [4,12]. Below, we provide an updated vision of the key aspects associated with the pharmacological and immunological properties of one such RNA therapeutic, the mRNA vaccines, and discuss the recent issues on their pharmacokinetics (i.e., distribution), pharmacodynamics (i.e., mechanism of action), and safety (i.e., reported adverse reactions). In addition, we overview the clinical trials having a mRNA vaccine as intervention which are registered on clinicaltrial.gov (accessed on 1 April 2023).

## 2. Methods

Pubmed (https://pubmed.ncbi.nlm.nih.gov/) free searches were performed on spring 2023. Articles containing the following keywords were considered for inclusion: “RNA-based drugs” or “mRNA vaccines” or “mRNA vaccines AND pharmacology” or “mRNA vaccines AND pharmacokinetics” or “mRNA vaccines AND immune response” or “mRNA vaccines AND safety”. Relevant articles were also identified from a manual search of reference lists within those included. The abstracts of the articles were screened to determine whether they should be included or excluded in the review. To be included, the article must have discussed original data, and been published in a peer-reviewed journal and written in English.

The clinical trials database clinicaltrials.gov (a searchable registry and results database of federally and privately supported clinical trials conducted in the United States and around the world) was utilized with the terms “mRNA” and “vaccine” in the search field of other terms. Results were filtered by hand, removing all those that were not actually relevant to mRNA vaccines. In particular, we found several entries referring to mRNA -engineered antigen presenting cells used for cancer therapy, which may represent a direct competitor of mRNA vaccines used for cancer therapy.

The remaining results were categorized, based on the objective of use. After a preliminary screening, we identified two major groups of medical conditions for which mRNA vaccines are being tested: cancer and viral infections. In each case, we reported the target disease and the state of advancement of the studies. For each disease, we also compared the relative prevalence of active mRNA vaccine studies in the database, over the total number of active studies.

## 3. mRNA-Based Vaccine Platform

The therapeutic strategies of RNA-based drugs using specifically mRNA molecules include different modalities such as the following: replacement therapy, where patients are treated with mRNA to supply therapeutic proteins or to compensate for a damaged gene/protein; cell therapy, where cell phenotype or function is changed by mRNA transfection, and then these altered cells are transplanted into the patient; and prophylactic/therapeutic vaccination, where mRNA encoding specific antigen(s) is administered to elicit protective immunity [5]. The medical promise of mRNA vaccines has been realized recently with the full approval of the Pfizer-BioNTech Comirnaty or Tozinameran (BNT162b2) and the Moderna Spikevax or Elasomeran (mRNA-1273). These two mRNA-based severe acute respiratory syndrome coronavirus (SARS-CoV)-2 vaccines were authorized approximately 11 months after publication of the viral sequence [13]. The transformative potential of the nucleic acid technology allowed the quick production of vaccines that contained the instructions for making a protein called the spike protein that is found on the surface of the SARS-CoV-2 virus, which causes COVID-19. Once the mRNA is injected into the body, it is taken up by cells in the muscle tissue near the injection site. The cells use the instructions in the mRNA to produce the spike protein, which is then displayed on the surface of the cells. Spike protein is recognized by the immune system as foreign, and the immune system mounts a response against it, producing antibodies and other immune cells that can recognize and neutralize the SARS-CoV-2 virus if it is encountered in the future.

mRNA vaccines have demonstrated their efficacy in the prevention of infectious diseases, although additional research is required to optimize their mRNA design, intracellular delivery, and applications beyond SARS-CoV-2 prophylaxis [13,14]. Further developments will also provide a new opportunity for protection against a variety of past and emerging infectious diseases [15]. In addition to SARS-CoV-2, mRNA-based vaccines against cytomegalovirus (CMV), Zika, human respiratory syncytial virus (RSV), influenza A (IAV), chikungunya virus (CHIKV), rabies (RBV), plasmodium species, or streptococci are currently under investigation in preclinical experiments or clinical trial [13,16,17,18,19,20,21,22,23]. It is noteworthy that researchers are also exploring applications other than infections. One area of investigation is the development of mRNA vaccines as a promising platform for cancer immunotherapy, since they are relatively easy to control and can be rapidly mass produced [14,24,25]. These therapeutic vaccines can be engineered to deliver mRNA that expresses full-length antigens containing multiple epitopes without major histocompatibility complex restriction, thus stimulating the immune system to recognize and attack cancer cells. Experiments are underway to test the safety and efficacy of mRNA vaccines for cancer treatment and encouraging results from early clinical trials with mRNA vaccines as monotherapy and in combination with checkpoint inhibitors have been obtained with different solid tumors for which therapy is still limited, including triple-negative breast cancer, ovarian cancer, non-small-cell lung cancer, colorectal cancer, pancreatic adenocarcinoma, melanoma, and gastrointestinal cancer, among others [15,26,27,28,29,30,31,32]. Allergy tolerization is another potential application as well [33,34].

Structurally, an mRNA vaccine consists of two major components: a targeted mRNA molecule encoding the antigen and its delivery system for intracellular release [15,35,36]. The synthetic mRNA produced by in vitro transcription (IVT) is endowed with a component similar to natural mRNA, namely a 5′ cap, a 5′ untranslated region (UTR), an open reading frame (ORF), a 3′ UTR, and a polyadenylated (poly(A)) tail [37]. Significant efforts have been employed to modify the structural elements of IVT mRNA in order to improve its intracellular stability and translational efficiency, therefore resulting in the production of high levels of the encoded protein over a longer timeframe (from a few minutes to more than 1 week) [35]. For instance, the insertion of 5′ and 3′ UTR elements from viral or eukaryotic genes, the addition of a synthetic cap or anti-reverse cap analogue and an optimal length of poly(A) and the optimization of the sequence/codons might greatly increase the half-life and expression of the mRNAs [15]. In addition, the mRNA sequence typically consists of modified nucleosides (i.e., pseudouridine, 2-thiouridine, 5-methyluridine, and 5-methylcytidine, among others) in order to address issues of stability and a detrimental immunogenicity [35]. Different delivery systems have been developed and optimized to enhance mRNA vaccine stability, biocompatibility, and homing to the desired cells and tissues. Inspired by the IVT systems, improving the safety profile, cationic lipids and polymers are now the most widely employed delivery tools for mRNA delivery [15]. Polysaccharide and dendrimers have been investigated as well, as have been lipid-based nanoparticles (LNPs); these are self-assembling particles mainly composed of cationic lipids, which may contain cholesterol and/or polyethylene glycol (PEG), depending on their formulation, with a diameter spanning between 80 to 200 nm. Such particles are uptaken via different endocytosis pathways (i.e., clathrin- or calveolin-mediated, lipid raft, or ApoE receptor-mediated mechanism) [38]. Once internalized, the mRNA escapes the nanoparticles by a not fully elucidated mechanism to then be in the cytoplasm, available to the translational cellular machinery [15,35,38].

As demonstrated for the anti-SARS-CoV-2 mRNA vaccines, once the mRNA molecule is delivered within the cell, it can be translated into the target protein of interest [35,36,39]. This potentially gives rise to a therapeutic whenever the treatment requires the expression of a target protein [39]. After translation, the target protein can induce both cell-mediated and antibody-mediated immune response [39]. The main features of such mRNA-based vaccines are a quicker cellular uptake, the lack of antigenicity of both the vector and the delivered IVT mRNA, the exploitation of a non-integrating, short-lifespan nucleic acidic molecule, and a “natural” processing by the cellular machinery, including protein folding and post-translational modification.

Overall, the pharmacology of mRNA-based vaccines differs from that of traditional vaccine approaches that use weakened or inactivated viruses or pieces of the virus to stimulate an immune response [40]. mRNA vaccine is superior to other conventional vaccine platforms due to high potency, safe administration, rapid development potentials, and cost-effective manufacturing. The mRNA in the vaccine breaks down quickly once it has been used to make the antigen protein. This helps to minimize any potential long-term effects of the vaccine on the body. Important issues are still there though and need to be solved: measuring the biodistribution of mRNA vaccines can be challenging due to the relatively short half-life of mRNA in the body and the need to detect low levels of mRNA in tissues [41,42]. In addition, delivering mRNA to the correct cells and tissues within the body can be a complex and challenging process [39]). From a regulatory point of view, the Pfizer-BioNTech and Moderna COVID-19 vaccines have faced a number of issues and challenges concerning safety and efficacy, storage and distribution, manufacturing and quality control, and vaccine hesitancy [42]. They have required close scrutiny and evaluation by regulatory agencies such as the European Medicines Agency (EMA) and the US Food and Drug Administration (FDA).

In line with this, WHO Expert Committee on Biological Standardization (ECBS) discussed and adopted the document “Evaluation of the quality, safety and efficacy of messenger RNA vaccines for the prevention of infectious diseases: regulatory considerations” (https://www.who.int/publications/m/item/annex-3-mRNA-vaccines-trs-no-1039), with the aim to provide information and regulatory considerations regarding key aspects of the manufacture and quality control, and nonclinical and clinical evaluation, of mRNA vaccines against infectious disease for human use. The following are some general features that should specifically be taken into account during the development and provided to the regulatory agencies when evaluating mRNA vaccines for their quality, safety, and efficacy:

i. The relevant biological characteristics of the specific mRNA technology used (e.g., the capability to trigger innate immune responses as well as target-antigen-specific responses: the quality, quantity, and bias of the immune responses and the in vivo stability);

ii. The rationale for the selection of the target antigen that is encoded and the rationale for any modification of the target antigen;

iii. The formulation of the final vaccine product and all excipients and the method of production of the delivery system, of any excipient, and of the final product;

iv. The toxicological and immunogenicity data on the delivery system and on any excipient.

The preclinical studies of candidate mRNA vaccines should be designed, conducted, and analyzed on a product-specific basis taking into account the intended clinical use. Particular attention should be paid to the durability of immune responses, biodistribution and persistence, RNA/LNP-induced inflammation, and unexpected and serious toxicities from modified nucleosides [43,44,45,46].

Clinical trials for each candidate mRNA vaccine must be designed to obtain as much as possible data about safety, immunogenicity, and efficacy, as expected for any other type of vaccine/drug, but with particular consideration given to potential concerns that may be more relevant for mRNA vaccines than for other types of vaccines as for instance: adverse immune effects and types and scope of immune responses [47,48,49].

## 4. Pharmacokinetic Considerations of mRNA Vaccines

In drug discovery and development, pharmacokinetic (PK) studies evaluate how the body interacts with administered medications with the intent to provide their time course and therefore to predict either their positive or negative effects in the body. The parameters analyzed through the measurement of medication blood concentration to determine its fate in the body are the processes of absorption, distribution, metabolism, and excretion (ADME) [50]. Acting together, these processes determine the onset, duration, and intensity of a drug’s effect; therefore, a comprehensive understanding of PK is essential in planning medication treatment regimen (e.g., dosing and timing of administration) and predicting the risk of possible adverse reactions [51]. Considering this, the regulatory agencies provide guidelines that require non-clinical and clinical PK assessment for the marketing authorization of most medicinal products.

The approval process for a vaccine is similar to that of other medications in most ways, passing through diverse non-clinical and clinical phases of research, with the notable difference that regulatory agencies do not require experimental preclinical and clinical PK studies for vaccine approval, following the recommendations of the World Health Organization (WHO) which do not consider studies on the biodistribution of the vaccine as a fundamental prerequisite for marketing authorizations (Guideline on Clinical Evaluation of New Vaccines EMEA/CHMP/VWP/164653/2005. https://www.ema.europa.eu/en/documents/scientific-guideline/guideline-clinical-evaluation-new-vaccines_en.pdf). [52] Of note, the WHO recommends that PK studies are unnecessary since most of the dose of the vaccine administered via intramuscular injection would remain in the muscle, while the rest would drain through the lymphatic system. The only exceptions for EMA are the cases in which new delivery systems are employed or when the vaccine contains novel adjuvants or excipients, such as the delivery system (https://www.ema.europa.eu/en/documents/scientific-guideline/note-guidance-clinical-evaluation-vaccines_en.pdf) and DNA vaccines utilizing novel vectors, formulations, methods of delivery, routes of administration for FDA (https://www.fda.gov/media/73667/download).

The approval of the first two mRNA-based COVID-19 vaccines from Moderna and Pfizer/BioNTech in 2021 opened new questions about the appropriateness of including PK studies in the marketing authorization process for this type of vaccines and for vaccines in general. Due to the particular nature of mRNA vaccines, many issues should be taken into account. Certainly, mRNA vaccines have a more pharmaceutical drug-like behavior than traditional vaccines. An mRNA vaccine can be assumed to be a prodrug since it is composed of a molecule, the mRNA, that needs to be converted into an active agent, the protein/antigen, to exert its pharmacological/immunological activity. Especially relevant is also the requirement for a delivery system to protect the mRNA and allow it to enter the target cells by endocytosis [53]. mRNA, indeed, is a large and negatively charged molecule that cannot pass through the anionic lipid bilayer of cell membranes. Furthermore, in the blood and tissues it is endocytosed by macrophages and dendritic cells and is rapidly degraded by nucleases [40]. Luckily, as mentioned above, several solutions, e.g., LNP, polyplexes and polymeric nanoparticles, peptides, and nanoemulsions, have been developed as delivery vehicles [13]. As such agents can most likely influence the behavior of a mRNA vaccine or the host’s responses to a vaccine, it is fundamental to assess their effects, including PK and pharmacodynamics, both separately from the mRNA and in combination with it [42,54]. Therefore, to provide sufficient data on the efficacy and security of vaccines, PK studies should include a quantitative evaluation of the injected mRNA-delivery system, of the antigen produced by the mRNA, and the antibody production [55]. In light of this, the time point of detection should also be set according to the kind of molecule being assessed, e.g., in terms of hours/days for mRNA-delivery systems, days for antigens, and weeks/months for antibodies [56]. However, to collect information that can be essential to the understanding of immune responses following vaccination, the biodistribution of mRNA and antigens should be performed also at longer periods (e.g., weeks/months).

As far as it concerns the administration route, intramuscular injection is so far the most common among all the type of vaccines [57], as it improves the immunogenicity of vaccinations and minimizes adverse reactions at the injection site. Muscles are, indeed, highly vascularized and are therefore good recruiters of antigen presenting cells at the site of injection [58]. Moreover, intramuscular injection offers high bioavailability by bypassing the harsh environment of the gastrointestinal tract and providing an easy entry into the bloodstream and the whole body [59]. However, the need for trained professionals to perform the injection, together with needle phobia are the two main issues with this type of vaccination. Therefore, alternative vaccine delivery methods, aimed at making the vaccination program more efficient and accessible, have been under investigation [60].

Analytical methods to study the ADME of mRNA vaccines also need to be designed and adapted. Indeed, not all the analytical assays established for traditional drugs/vaccines, for instance mass spectrometry-based assays (the gold standard for small molecule drugs, metabolites, or carrier components), are still applicable to this new type of molecule, requiring new technology and specific assays/regulatory standards to be developed [61]. Moreover, the analytical techniques should be validated, to demonstrate that they are appropriate for detecting the target (i.e., the mRNA, the delivery system, and the expressed protein) at the molecular level and in the relevant biological matrix [42]. For preclinical biodistribution studies, regulatory frameworks indicate different techniques that can be employed for the detection of the mRNA and the expressed proteins, e.g., reverse transcription qPCR (RT-qPCR), enzyme-linked immunosorbent assay (ELISA), multiplex branched DNA assay, immunohistochemistry (IHC), western blot, in situ hybridization (ISH), digital PCR, flow cytometry, and in vitro and in vivo imaging techniques (International Council for Harmonisation of Technical Requirements for Pharmaceuticals for Human Use, DRAFT: ICH guideline S12 on nonclinical biodistribution considerations for gene therapy products (EMA/CHMP/ICH/318372/2021), 2021) [42].

The PKs of mRNA vaccines approved for COVID-19 have been studied in rodents and non-human primates. For both Moderna and Pfizer/BioNTech vaccines no dedicated ADME studies with the candidate mRNAs (BNT162b2 and mRNA-1273) have been conducted to receive conditional authorization, but the preclinical biodistribution of the vaccine platforms was evaluated by whole-body autoradiography, liquid chromatography-mass spectrometry (LC-MS), or hybridization assay for formulations containing the proprietary LNP delivery systems and surrogate mRNAs (Assessment Report EMA/707383/2020 Corr.1*: Comirnaty COVID-19 MRNA Vaccine (Nucleoside- Modified); https://www.ema.europa.eu/en/documents/assessment-report/comirnaty-epar-public-assessment-report_en.pdf; Assessment Report EMA/15689/2021 Corr.1*: COVID-19 Vaccine Moderna; https://www.ema.europa.eu/en/documents/assessment-report/spikevax-previously-covid-19-vaccine-moderna-epar-public-assessment-report_en.pdf). This was in accordance with the regulatory guidelines and with the biologically plausible hypothesis that the distribution of mRNA vaccines is determined by the LNP content, whereas the influence of the mRNA itself should be very limited. Intramuscular injection of the vaccines leads to an initial accumulation at the injection site, after which LNPs are rapidly transported to proximal lymph nodes (LNs) or reach systemic circulation and may be targeted at the liver, spleen, or other organs [13]. For BNT162b2 and mRNA-1273 data obtained in mice and rats demonstrated a distribution primarily in the liver and in LNs and spleen, respectively, within few hours from the injection. The half-life of mRNA was estimated for the Moderna vaccine at the site of injection (~15 h, as mean value), proximal popliteal and axillary distal lymph nodes (~30 h), and spleen (~63 h) (Assessment Report EMA/707383/2020 Corr.1*: Comirnaty COVID-19 MRNA Vaccine (Nucleoside- Modified); https://www.ema.europa.eu/en/documents/assessment-report/comirnaty-epar-public-assessment-report_en.pdf). For BNT162b2, the applicant measured the biodistribution of both the LNPs and the protein product of the mRNA (luciferase modRNA). The LNP excipient ALC-0315 was eliminated slowly from the liver and after 2 weeks the concentration was still ~25% of the maximum concentration with a complete elimination expected after 6 weeks. The luciferase signal in the liver peaked at 6 h post injection and decreased to background levels 48 h after injection (European Medicines Agency. Assessment Report EMA/15689/2021 Corr.1*: COVID-19 Vaccine Moderna; https://www.ema.europa.eu/en/documents/assessment-report/spikevax-previously-covid-19-vaccine-moderna-epar-public-assessment-report_en.pdf). In general, the mRNA translation is estimated to occur rapidly, from hours to a day and to span up to 10 days [62]. Although the validation of analytical techniques is a fundamental issue for authorized mRNA therapeutics, the choice of animal species and animal model, the number of animals, the duration of longitudinal animal studies, and the panel of tissues to be examined, as well as the compliance to good laboratory practice have a key role to appropriately study the preclinical biodistribution of these drugs [42]. Notably, recent studies have investigated the biodistribution and functional half-life of vaccine-associated S-protein mRNA in vaccinated healthy individuals and in patients [63,64,65], finding that both BNT162b2 and mRNA-1273 can remain in systemic circulation for at least 2 weeks, with the ability to induce S-protein expression once having entered into susceptible cells and tissues and to boost the immune system. Although these studies suffer several limitations, including their small sample size, these findings support the idea that mice, and even non-human primates, do not reliably predict human PK features of mRNA vaccines. This suggests the necessity of human PK studies before the market authorization to employ mRNA vaccines under the best conditions of efficacy and safety.

## 5. Mechanism of Action of mRNA Vaccines: Antigen Presentation

**Figure 2:**
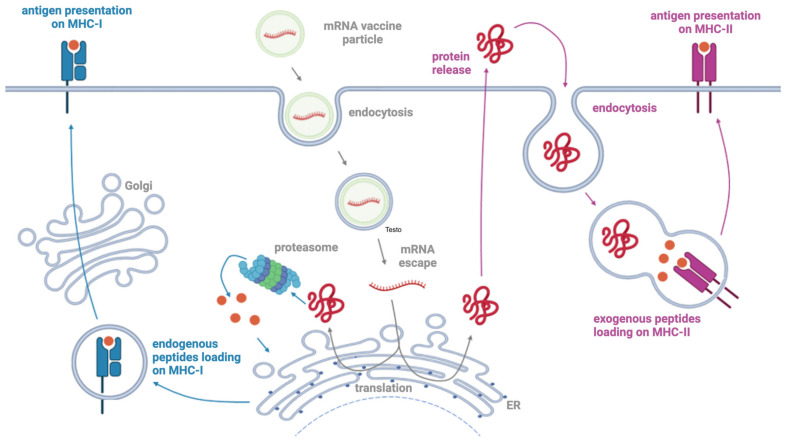
Scheme of the mechanism of action of mRNA vaccines. The mRNA encapsulated in the LNP enters the cell via endocytosis and is translated into the antigen by the cellular translation machinery. The antigen is processed by the proteasome system into endogenous peptides that can be presented by MHC-I molecules on the cell surface for the recognition by CD8+ cytotoxic T cells. The antigen can also be released by the cell and endocytosed by antigen-presenting cells for presentation by MHC-II molecules and recognition by CD4+ T helper cells. Created with Biorender.com.

mRNA vaccines act on cells through different and more sophisticated ways than conventional vaccines [15,66]. The latest, being based on either inactivated or live-attenuated pathogens, are recognized by the immune system as extracellular antigens and therefore elicit the MHC-II-mediated antigen presentation, regardless of the pathogen. Moreover, inactivated or live-attenuated conventional vaccines are based on the whole pathogen. More modern vaccines can be based on a single recombinant protein or oligosaccharide. In this line, mRNA vaccines only encode for a specific protein, either the whole sequence or a single domain. By choosing the antigen or even the epitope, modern vaccines can shape the immune response against a specific target region that can have a neutralizing function or that it would not efficiently result as immune dominant. mRNA vaccines must be delivered within the cells to be translated into antigens. Then, such newly translated antigenic protein will be presented to immune cells by both MHC class I and II [35] (Figure 2). Indeed, the antigen can be secreted and in turn be uptaken by cells as an exogenous antigen, thus eliciting MHC class II antigen presentation. Alternatively, the antigen can be processed by intracellular machinery as endogenous antigen, thus eliciting MHC class I-activated immune responses. Antigens presented by MHC class I trigger the cytotoxic CD8+ T cell-mediated cellular immunity, greatly enhancing vaccine efficacy. This kind of immune response cannot be achieved by conventional inactivated or live-attenuated vaccines. However, cell-mediated immunity is relied upon by the anti-viral (and anti-tumor) immune response, as viral (and tumor) antigens are produced within the cell as endogenous proteins. In addition, mRNA-based vaccines are recognized by pathogen recognition receptors (PRR) and therefore boost the innate immune response. They have been defined as self-adjuvating vaccines for this very reason [39]. As mRNA vaccines are based on modified nucleosides, such self-adjuvating properties are thought to rely on the LNP rather than the mRNA molecule.

### 5.1. Innate Immunity

Intramuscular administration of LNP-based nucleoside-modified mRNA vaccines results in local inflammation. By triggering the production of cytokines, chemokines, and other inflammatory mediators, neutrophils, monocytes, dendritic cells (DCs), and various immune cells from the blood to the injection site are recruited. The LNP carrier is believed to be the main driver of the self-adjuvant functions, as it can trigger by itself secretion of CCL2, CCL3, CCL4, GM-CSF, CXCL2, CXCL10, IL1β, IL6, TNFα, and IFNγ, among others [67,68]. As mentioned above, it has been reported that the size and surface properties of the particles can impact biodistribution, protein absorption, and cellular uptake [67]. Moreover, a role for opsonization has been proposed that would tune the receptor-mediated uptake of such particles, enhancing particles’ delivery to the draining lymph node [67,69]. Once uptaken by immune cells, mRNA vaccines are typically translated into protein within such cells. While the expression in neutrophils appears to be dimmed [70], monocytes/macrophages and DCs are efficient at expressing the vaccines’ antigen and then migrating to the draining lymph node and exhibiting an increased expression of co-stimulatory markers (i.e., CD80/CD86).

The incorporation of modified nucleosides (e.g., pseudouridine and its derivatives instead of uridine) drastically lowers the recognition of mRNA by toll-like receptors (TLR) or other RNA sensors, which would otherwise lead to vaccine mRNA destruction, resulting in reduced inflammation and greatly improved translation [71]. In a variety of cells, including immune cells, fibroblasts, and epithelial cells, TLR3, TLR7, and TLR8 are expressed in the endosomal intracellular compartment. TLR3 recognizes double-stranded RNA (dsRNA), whereas TLR7 and TLR8 single-stranded RNA (ssRNA). Such receptors are activated by endocytosed mRNA and in turn induce interferon (IFN) response and type 3 inflammasome activation [67,72]. Although acting as a self-adjuvant by promoting innate immunity, this would lead to negative effects on antigen protein expression, lowering vaccine efficiency and effectiveness [35].

### 5.2. Adaptive Immunity

Once they have migrated to the draining lymph node, monocytes/macrophages and DCs proceed to antigen presentation and lymphocytes priming. In particular, CD4+ T helper cells (Th), including T follicular helper (Tfh), which in turn provide support for B cells activation in the germinal centers, and CD8+ cytotoxic T cells (CTL) [73,74]. Confirming observations in preclinical animal models [48,75,76], clinical trials reported that mRNA vaccines induce a strong secretion of type 1 (Th-1) cytokines (i.e., IFNγ, IL2, and TNFα) rather than type 2 (Th-2) (i.e., IL4, IL5, and IL13), indicating a Th1-skewed immune response, which is the cell-mediated immune response, typically predominant in viral infections and cancers [48]. Together with Th1 cells, Tfh cells are strongly induced by mRNA vaccines, as per the expression of their canonical markers (i.e., IL21 and CD40L) [77]. These cells are crucial for B cell activation, B cell isotype switching and generation of long-term memory cells in the B lymphocytes compartment. IFNγ-producing CTLs were enhanced by mRNA vaccine as well, although discrepant results were obtained with different mRNA vaccines: indeed BNT162b2 elicited a strong CTL response, while mRNA-1273 did not [35,78].

As the natural SARS-CoV-2 infection induces potent antibody (Ab) production in convalescent individuals, mRNA vaccines were developed to induce robust Ab responses [48]. Also, once the SARS-CoV-2 neutralizing domain was identified, vaccines were designed to induce a high neutralizing antibody (nAb). Contrary to what has been observed for SARS-CoV-2-specific T lymphocytes, B cell response is waning overtime, decreasing the plasmatic titer of more than 60% in a 3–4 month span [79,80]. The low titer of neutralizing antibody correlates with a higher re-infection rate and vaccine breakthrough cases [81,82]. Moreover, nAb efficacy is reduced by the emergence of new variants, drastically for some of the variants of concern (VOC), although never completely abrogated [83,84]. Vaccine breakthrough infections were reported to boost nAb plasmatic titer, as well as second/booster vaccine doses, that had a cross-reactive protective effect against all known variants [85].

Overall, the complete mRNA vaccination plan is proved to provide protection from infection and progression to disease, by generating long-lived memory B cells and plasma cells, together with helper and cytotoxic T cells. As the activation of lymphocytes response depends on the number of antigenic determinants, vaccines based on the full-length Spike mRNA sequence can elicit a greater immune response compared to the shorter RBD-only vaccine. The number of epitopes contained in the full-length Spike protein is greater than those in the RBD-only. Additionally, while the vast majority of CD4+ T cells recognize epitopes in any Spike protein domains, the majority of CD8+ T cell response is directed at epitopes in the N-terminal domain only [86].

### 5.3. Mucosal Immunity

As a respiratory virus, SARS-CoV-2 is mainly transmitted through exposure of the upper airway mucosa to infected secretions. Thus, oral mucosal immunity plays a pivotal role in early defense against SARS-CoV-2 [87]. There has been considerable interest in achieving localized immune responses in a way that provides better protection in the respiratory mucosa at the site of virus entry, in contrast to traditional vaccine approaches that focus on systemic immunity only. Typically, vaccines administered both via intramuscular injection or via mucosal exposure elicit systemic plasmatic IgG Ab, while mucosal IgA titers tend to be modest and variable in the first case. Anti-SARS-CoV-2 mRNA vaccines succeeded to elicit mucosal SARS-CoV-2-specific IgA. Overall, the salivary titer and the neutralizing activity were lower than the plasmatic ones. Natural infection was able to induce consistently higher titer than vaccines [87,88,89,90,91], although booster doses increased the titer of salivary IgA as well. Finally, mRNA vaccine administered by intramuscular injection succeeded in eliciting mucosal protection but some alternative vaccinal strategies have been also proposed. For instance, in rodent animal models an efficient mucosal SARS-CoV-2-specific IgA production was elicited by booster doses administered intranasally [92].

### 5.4. mRNA- vs. DNA-Based Vaccines

Some of the features of mRNA vaccines are also present in DNA vaccines. Indeed, vaccines encoded by nucleic-acid sequences enable a precise antigen design, resulting in stabilized and more immunogenic conformations. Different strategies can be achieved by carefully selecting specific key antigenic epitopes or by fusing different sequences in a single vaccine. The resulting protein is a “native-like” protein bearing all the necessary post-translational modifications and foldings as it is produced intracellularly [37]. Moreover, both mRNA- and DNA-based vaccines, compared to conventional inactivated pathogens, protein subunits, and peptide vaccines, which predominantly stimulate antibody responses, are able to induce both humoral and cell-mediated responses due to the expression of encoded antigens in host antigen presenting cells (see above). mRNA-based vaccines, however, have multiple specific advantages over the DNA-based ones. mRNA can be easily synthetized to encode for any kind of protein antigen and the mRNA enclosed within mRNA-based vaccines can be adapted to a vast plethora of pathologies, including emerging epidemics and cancers, exploiting the same manipulable platforms [93]. In addition, cytoplasmic mRNA is efficiently and directly traduced to a cell functional protein, while DNA needs to travel to the nucleus in order to be transcribed, lowering efficiency [94]. DNA-based vaccines exploit viral vectors as delivery system, which elicit a long-lived immune response against the vector’s viral antigens, leading to vaccine destruction and in turn lowering the efficacy of subsequent vaccine administration, whereas another advantage of mRNA/LNP vaccines is self-adjuvating property. This contributes to promote robust long-lasting immune responses, in contrast to to protein-based vaccines, which are usually coupled with additive adjuvants [95]. Not to be underestimated, mRNA molecules have a short span of life, even with nucleoside modifications. Such transient activity allows the vaccine mRNA to be cleared, while DNA-based vaccines are considered to be more stable, increasing the risk of integration as well [23]. In this respect, mRNA vaccines show several important advantages in terms of safety such as no risk of integration into the host DNA and no potential risk of infection or mutagenesis [96]. Additionally, since production enables short development times, safety concerns due to the presence of cell-derived impurities and contaminating microorganisms (commonly found in other platforms) are minimized [15].

## 6. Safety Profile of mRNA Vaccines: Evidence from Clinical Trial Data and Post-Market Surveillance

In line with the low theoretical risk for safety concerns, the approved vaccines have shown acceptable safety profiles during their evaluation in clinical trials: the side effects most frequently reported included heat, pain, redness, and swelling at the injection site [78,97]. Other systemic side effects reported were fatigue, fever, headache, myalgias, and arthralgias, with most occurring within 1 to 2 days following vaccination and lasting between 24 and 48 h [78,97]. Hypersensitivity adverse side effects were reported in both the placebo and vaccine groups in both trials. The incidence of systemic side effects was less than 1% following the first vaccine dose and less than 2% following the second dose, with the exception of fatigue (3.8%) and headaches (2.0%). The younger vaccine recipients (between 16 and 55 years of age) reported systemic events more frequently than their older counterparts (over 55 years of age) [78,97], maybe due to a more robust immune response in younger individuals compared to the older population.

Regarding serious adverse events, vaccine administration-related shoulder injury, right axillary lymphadenopathy, paroxysmal ventricular arrhythmia, and right leg paresthesia were reported. None of the deaths (due to arteriosclerosis and cardiac arrest) were found to be connected to the vaccine [78,97].

### 6.1. Data from Post-Marketing Studies

Safety profiles of mRNA-based vaccines in clinical development are acceptable as they are well tolerated; however, some mRNA-based vaccine platforms induce potent type I interferon responses, raising potential safety concerns including inflammation and autoimmunity worthy of further evaluation [98]. Additionally, the presence of extracellular RNA represents a risk factor for promoting blood coagulation and pathological thrombus formation [99], suggesting the need to better characterize potential safety issues related to previously unknown or potential long-term effects.

Data from post-approval studies is always critical to evaluating the safety of approved drugs and vaccines but especially during the COVID-19 pandemic. Monitoring the safety profile of vaccines has been of paramount importance to detect rare serious and serious unknown events following immunization, thus ensuring guaranteed patient safety. Additionally, the large and rapid pace of the COVID-19 vaccination campaigns did not allow full identification of risk groups for developing adverse events following immunization (AEFIs), supporting the need for further research. In line with this evidence, starting from 2021, there has been an increasing number of post-marketing studies (mainly including cohort studies based on medical claims or electronic health records and disproportionality analyses using spontaneous adverse event reporting systems) aimed at better characterizing the safety profile of mRNA vaccines both in the general population as well as in fragile subjects such as children and adolescents, elderly people, and pregnant women, who are usually excluded from clinical trials and for whom safety and efficacy data are lacking.

Importantly, the data from naturalistic studies are reassuring [100,101]. Mild anaphylactic reactions have been seen in 4.7 per million COVID-19 vaccinations: in 2.5 per million vaccinations with the Moderna vaccine and 2.2 per million with Pfizer–BioNTech vaccine [102]. The higher risk for these allergic responses seen with COVID-19 vaccinations compared to traditional vaccines [103] might attributed to pre-existing antibodies against the PEGylated lipids (formed in the body in response to the presence of PEG in many consumer products, such as toothpastes and shampoos) which are used in lipid nanoparticles (LNPs) for mRNA vaccine delivery. It has been supposed that PEG may activate humoral immunity in a subset of the population in a T cell-independent manner, by directly crosslinking the B cell receptor and introducing IgM production [104]. Anti-PEG antibodies are reported in 40% of the population, which can accelerate and heighten the risk of allergic reactions and negatively impact on the vaccine efficacy [105]. Based on this evidence, patients with severe allergies or immediate reaction during 4 h. to PEG and its derivatives should not be vaccinated with Pfizer-BioNTech or Moderna mRNA vaccines, according to the CDC recommendation [106].

### 6.2. Adverse Events of Special Interest

In both Pfizer and Moderna clinical trials, coagulation disorders have been of central concern for mRNA vaccines. As reported by the FDA in July 2021, adverse events of special interest (detected by suing medical claims data) that occurred in older Americans included pulmonary embolism, acute myocardial infarction, immune thrombocytopenia, and disseminated intravascular coagulation (https://www.fda.gov/vaccines-blood-biologics/safety-availability-biologics/initial-results-near-real-time-safety-monitoring-covid-19-vaccines-persons-aged-65-years-and-older). The first case on immune thrombocytopenia was reported in a 22-year-old patient suffering from gum bleeding and petechiae after vaccination with Comirnaty in January 2021; however, it was very difficult to exclude alternative causes and to clearly attribute a direct role of the vaccine to the event [107]. Deep vein thrombosis following the second dose of Pfizer COVID-19 vaccine was officially confirmed in one case after one month [108]. The occurrence of acute deep vein thrombosis was also reported 3 days after the second dose of Moderna vaccination in a 27-year-old Caucasian female [109]. A clotting event was reported also in a 60-year-old man who experienced thrombocytopenia and purpuric after being vaccinated by Moderna COVID-19 vaccine [110]. Such cases can be purely coincidental [111] and are presumably caused by the production of antibodies against PF-4 [112] following the mRNA COVID-19 vaccines.

Compared to the influenza vaccine, an excess risk for coagulation events, hemorrhages, and thromboses [113] was found for mRNA COVID-19 vaccines, as reported in a pharmacovigilance study using VAERS and EudraVigilance including more than 7.8 million adverse reactions of about 1.6 million persons. These numbers must be taken with some caution as the spontaneous reporting system database collected reports that do not represent conclusive evidence of a causal association between vaccine exposure and adverse reactions and for which the reporting activity is influenced by the well-known phenomena of under or over reporting, due to public awareness of certain reactions.

With regard to adverse events of special interest, starting from April 2021, increased cases of myocarditis and pericarditis shortly after vaccination with Pfizer-BioNTech and Moderna vaccines were reported in several countries including Asia, Europe, the Middle East, and North America [114,115,116,117,118,119,120,121]. Since then, many observational studies replicated previous findings among younger individuals [122] by using a variety of real-world data analyses including comparisons of observed-to-expected rates, case–control studies, self-controlled cases series, and cohort studies. Higher-than-expected rates of myocarditis and pericarditis were found in young individuals, with the highest risk among men aged 18–25 years following their second COVID-19 mRNA vaccine dose [123]: the incidence rate within 1–7 days of vaccination was 2.17 (95% CI 1.55–3.04) cases per 100,000 person-days for the Moderna vaccine, and 1.71 (1.31–2.23) cases per 100,000 person-days for the Pfizer-BioNTech vaccine. However, important study limitations should be taken into account. As stated by the CDC, data from multiple studies show a rare risk for myocarditis and/or pericarditis following receipt of mRNA COVID-19 vaccines. These rare cases of myocarditis or pericarditis have occurred most frequently in adolescent and young adult males, aged 16 years and older within 7 days after receiving the second dose of mRNA COVID-19 vaccine (https://www.cdc.gov/vaccines/covid-19/clinical-considerations/myocarditis.html). Currently, the long-term outcomes of vaccine-associated myocarditis and pericarditis are not fully clarified; however, the available evidence on the short-term clinical outcomes are reassuring. In many cases, people manifesting myocarditis and pericarditis following vaccination with mRNA COVID-19 vaccines responded well to treatments, with significant improvements in symptoms (https://www.cdc.gov/vaccines/covid-19/clinical-considerations/myocarditis.html).

It is worth mentioning that other vaccinations, especially smallpox vaccination and also modified Vaccinia Ankara vaccine for Mpox, have been associated with a similar increased risk of cardiovascular adverse events, including myocarditis [124,125]. These findings suggest that the disease mechanism is specific neither to the newly developed mRNA vaccines nor to exposure to the SARS-CoV-2 spike protein. Other mechanisms have been suggested; however, the clear underlying mechanism explaining the association has not been fully elucidated. Future mechanistic studies into potential mechanisms are therefore warranted to provide valuable insights.

### 6.3. Care of Special Populations

#### 6.3.1. Children and Adolescents

In special populations, such as children and adolescents, usually excluded from clinical trials, and for whom safety and efficacy data are lacking, findings are also promising [126]. Over 26.6 million COVID-19 vaccinations were given to 0.22 million people aged between 5 and 17 years as of February 2022 in the United States. In 2021, the first systematic review of high-quality RCTs on the safety and efficacy of mRNA vaccines against COVID-19 in participants aged 5–17 years old included two studies on the BNT162b2 vaccine and one study on the mRNA-1273 vaccine. The authors found no serious vaccine-related adverse events [126], and the most common local and systemic events included injection site pain, fatigue, and headache. More in detail, in the multinational, placebo-controlled trial by Frenck et al., a total of 2260 adolescents (age range: 12 to 15 years) received injections; 1131 received BNT162b2, and 1129 received placebo [127]. BNT162b2 was highly effective and had a favorable safety profile, with mainly transient mild-to-moderate adverse reactions including injection-site pain, fatigue, and headache, with similar rates of overall AEFI detected in the BNT162b2 vaccine (6.0%) and placebo (5.9%) groups [127].

In line with this overview, in data from the phase 2–3 trial by Walter et al. [128], in which a total of 2268 children were randomly assigned to receive the BNT162b2 vaccine (1517 children) or placebo (751 children), the vaccine had a favorable safety profile, and no vaccine-related serious adverse events were reported. In the mRNA-1273 vaccine group, Ali et al. [129] found a significantly increased incidence of adverse reactions after vaccination in mRNA-1273 vaccine recipients (Dose 1: 95.9%; Dose 2: 97.1%) compared with placebo recipients (Dose 1: 65.1%; Dose 2: 55.7%). Despite the fact that each included study showed a higher risk of local and systemic events for mRNA vaccine recipients compared to the placebo, most adverse events were mild and typically resolved within 4 days. Then, in 2023, in the last systematic review and meta-analysis including 17 studies with 10,935,541 vaccinated and 2,635,251 unvaccinated children aged 5 to 11 years, the overall frequency of severe AEFI, including myocarditis, was low [130].

#### 6.3.2. Elderly

Data from a recent meta-analysis of RCTs confirm that the vaccination in the elderly is safe and effective [131]; common local and systemic previously known AEFI were reported including pain, itching, redness at the injection site, fever, chills, nausea, headache, diarrhea, and joint pain. The authors found a higher incidence of AEs in younger individuals than older adults (OR = 0.42, 95% CI (0.31, 0.56), *p* < 0.01), likely due to the immune senescence (i.e., the gradual deterioration of the immune system due to aging), which leads to a lower incidence of adverse reactions in the elderly.

More recently, a nationally representative early warning system based on the US Centers for Medicare & Medicaid Services (CMS) was used to monitor special outcomes of interest after COVID-19 vaccination in elderly persons. Data included 30,712,101 elderly persons and a total of 34,639,937 doses. The following four events met the threshold for a statistical signal following BNT162b2 vaccination: pulmonary embolism (PE; RR = 1.54), acute myocardial infarction (AMI; RR = 1.42), disseminated intravascular coagulation (DIC; RR = 1.91), and immune thrombocytopenia (ITP; RR = 1.44). No statistical signals were identified following vaccination with the mRNA-1273 [132]. No firm conclusion can be drawn; in view of the observational nature of the study, more robust epidemiologic studies with adjustment for confounding, are warranted to further evaluate these signals and clarify the role of the vaccines in causing these outcomes.

#### 6.3.3. Pregnancy

When the COVID-19 vaccination campaign started, efficacy and safety data on mRNA vaccines in pregnancy were limited because of the exclusion of pregnant people from pre-authorization clinical trials, with a consequent low rate of coverage vaccination among this population. Worldwide research groups have been contributing to address potential safety issues in order to support and enhance the uptake of COVID-19 among pregnant women. Tackling the vaccine hesitancy in pregnancy is indeed particularly important given the increased rate of significant complications related to COVID-19 and because there are not currently any vaccines available for infants younger than 6 months. The growing body of evidence on the safety profile of mRNA vaccines during pregnancy is reassuring [133]. Findings from a recent observational cohort study involving pregnant and non-pregnant females aged 15–49 years showed that the most common significant AEs following both doses of mRNA COVID-19 vaccines in pregnant people were malaise or myalgia (66 [3.5%] of 1892 for two doses of BNT162b2 and 139 [11.4%] of 1216 for two doses of mRNA-1273) and headache or migraine (41 [2.1%] of 1892 for two doses of BNT162b2 and 103 [8.5%] of 1216 for two doses of mRNA-1273). Serious events resulting in emergency department visit or hospital admission in the previous 7 days were rare (<1.0% in all groups) [134]. Similar studies based on spontaneous reporting system databases, such as the vaccine adverse event report system (VAERS), provide reassuring evidence that COVID-19 mRNA vaccines are safe for pregnant women and their infants. No serious safety alerts neither increased risk of negative neonatal outcomes was found [135].

Consistently with the systemic reactogenicity profile of mRNA vaccines, the commonest non-pregnancy adverse events included headache (482; 2.21%), fatigue (472; 2.16%), and pyrexia (436; 2.00%), while the highest reporting rate of adverse pregnancy outcomes were abortions spontaneous (762; 3.49%), and vaginal hemorrhage (229; 1.05%) [136]. However, due to the well-known intrinsic limitations of studies based on passive surveillance system (low quality data, missing information, underreporting, overreporting, confounding bias, etc.), it is impossible to establish a causal correlation between the reported events and COVID-19 vaccination. In line with this overall encouraging evidence, a recent meta-analysis of 18 observational studies showed that there was no significant impact of COVID-19 vaccination (vs. no vaccination) on the odds of preterm birth before 37 weeks’ gestation (37,195 vaccinated vs. 369,924 unvaccinated, P = 0.269, I2 = 96.8%) and of miscarriage (pooled OR 1.00; 95% CI 0.92–1.09, 15,684 vaccinated vs. 108,249 unvaccinated population, P = 0.988, I2 = 19.8%) [137].

## 7. Relevant Clinical Trials on mRNA Vaccines

The clinical trials database clinicaltrials.gov was searched in spring 2023, using the terms “mRNA” and “vaccine” in the search field of other terms. Results were filtered by hand, removing all those that were not actually relevant to mRNA vaccines. In particular, we found several entries referring to mRNA-engineered antigen presenting cells used for cancer therapy, which may represent a direct competitor of mRNA vaccines used for cancer therapy. Remaining results were categorized, based on the objective of use. After a preliminary screening, we identified two major groups of medical conditions for which mRNA vaccines are being tested: cancer and viral infections. In either case, we reported the target disease and the state of advancement of the studies. For each disease we also compared the relative prevalence of mRNA vaccine active studies in the database, over the total number of active studies. Our search found 450 entries; of these, five were excluded for being not relevant. Another 29 studies were excluded from the main results since they used various techniques to load antigen-presenting cells with mRNA often ex-vivo; for instance, they could be transfected or infected with viral vectors, bearing constructs coding for cancer neoantigens. Engineered antigen-presenting cells have the purpose of triggering an immune response against cancer cells. Strategies used in this field of study are various and they have been reviewed recently [138].

We thus collected 416 studies dealing with mRNA vaccines, predominantly used against viral infections in several studies to trigger an immune response against cancer cells, and in minimal part for other infections (Table 1). SARS-CoV-2 viral infections were the predominant field of research, covering 79.8% of all studies on mRNA vaccines; of these studies, 71.7% were active and 21.7% completed. Considering viral infections besides SARS-CoV-2, the second and third most targeted were those by influenza virus (14 studies) and cytomegalovirus (7 studies); mRNA vaccines also stand out as treatments under study for respiratory syncytial virus (6 studies) and HIV (5 studies) (Table 1). For these studies, 66.7% were active and 29.7% completed, indicating that this field of research can still be considered novel regardless of the target disease. As a comparison, only 29.7% of the studies we found on engineered antigen presenting cells were active, indicating a more mature field of clinical research.

When comparing the prevalence of mRNA vaccine studies over vaccine active studies (Table 2), the dearth of studies on alternative approaches becomes evident with respect to Nipah and Zika viruses, for which mRNA studies are, respectively, 50% and 33.3%, due to a lack of ongoing studies overall. Other important fields of prevalence of mRNA vaccines over vaccine active studies are cytomegalovirus (29.4%), Epstein–Barr virus (16.7%), and respiratory syncytial virus (14%), for which there is also a sizeable number of studies, and of course SARS-CoV-2 (26.1%). Overall, we can observe that mRNA vaccines are more prevalent among studies when dealing with highly variable or scantly immunogenic viruses for which the prevalence of mRNA vaccines is mainly determined by the low number of studies on other approaches; although these cannot be considered orphan diseases, mRNA vaccines represent a more feasible approach as compared to traditional vaccine therapies.

In the field of cancer vaccines, mRNA approaches deal with a variety of competitive approaches, from engineered antigen presenting cells, to peptide vaccines, to CAR-T and more. Indeed, the most abundant studies concern melanoma and glioblastoma (six each), followed by advanced solid cancer (five studies), but the overall prevalence among mRNA vaccine active studies is remarkable only for Epstein–Barr related cancer (only two active studies, both with mRNA vaccine), considerable for advanced solid cancer (14.8%) and melanoma (10.8%), and scant or absent for other applications. It appears that the multitude of alternative treatments in the field of cancer impacts adversely on the use of mRNA vaccines. One possible explanation is that in this peculiar field, the direct loading of antigen presenting cells, carried out both ex vivo and directly in homo, may represent a more efficient alternative to triggering a response with intramuscular mRNA vaccine injections. When a higher number of studies on mRNA vaccines are completed, it would be interesting to compare their efficacy versus antigen presenting cells engineering approaches.

### Phase 2/3 Trials

A few studies are phase 3 and 2 randomized controlled trials (Table 3), and thus deserve specific consideration; most phase 1 studies have been already reviewed elsewhere [96] and will not be examined here in detail.

Cytomegalovirus is not currently targeted by approved vaccines, although there are effective antivirals. While the clinical course of cytomegalovirus infection is often benign, the availability of a fully effective vaccine against cytomegalovirus is of high importance for patients who received allogenic transplantation of hematopoietic cells, as in this context cytomegalovirus may severely hit lungs and the gastrointestinal tract [139]. A candidate vaccine based on a recombinant glycoprotein B obtained partial efficacy, with neutralizing antibodies present in around 50–70% of treated subjects depending on the chosen adjuvants and on the conditions of use [140,141,142], which leaves room for improvement through mRNA vaccines. The mRNA vaccine mRNA-1647 was tested in two phase 2 and one phase 3 studies. NCT04232280 tested three different doses of mRNA-1647 versus placebo, in 315 healthy adults with ascertained cytomegalovirus seropositivity or negativity. The treatment consisted of three injections at 0, 2, and 6 months. This study, which included safety as well as efficacy outcomes (seroconversion), has been recently completed and results are not yet available. NCT05085366 is a phase 3 study following the promising interim analysis of the phase 2 study. It is currently recruiting and plans to use one fixed dose of mRNA-1647 versus placebo in healthy females who tested seronegative for cytomegalovirus. The treatment consists of three injections at 0, 2, and 6 months. The main outcome will be seroconversion against antigens not encoded by the mRNA-1647, indicative of a mature immune response. The study is expected to be completed by April 2026. NCT05683457 is a phase 2 trial just about to start recruitment; it will use one fixed dose of mRNA-1647 versus placebo, in patients who received allogenic transplantation of hematopoietic cells. Administration will be at 42, 67, 92, and 180 days after withdrawing antiviral prophylaxis and the main outcome will be the time to occurrence of clinically evident cytomegalovirus disease or initiation of antiviral therapy up to 9 months after hematopoietic cells transplantation; the study will presumably end in August 2025.

Influenza virus can infect about one child in five and one adult in ten in the community when considering the scant prevention measures of the pre-covid era [143], and symptomatic influenza can be a medical threat to fragile patients and causes around 290 to 650 thousand deaths per year [144]. Vaccines against influenza currently available are based on inactivated virus or recombinant antigens and have an efficacy of about 18% to 61% based on the virus lineage and number of shots taken; interestingly, repeated vaccination with approved products decreases vaccine effectiveness over time [145]. Considering this relative lack of efficacy, two candidate mRNA vaccines, mRNA-1010 and qIRV (22/23), are being developed in competition. Of these, more advanced in clinical development is mRNA-1010, which was tested in the small phase 3 study NCT05415462 against the licensed quadrivalent inactivated seasonal influenza vaccine, both used in a single dose and shot, in 6102 subjects aged 18 years and older. Main outcomes comprised seroconversion of anti-hemagglutinin antibodies and antibody titer, besides safety; study will be completed by August 2023. The phase 3 study NCT05566639 is testing the same treatments in 23,000 subjects, focusing on the age 50 years and older, and has the main outcomes of influenza-like illness caused by an influenza strain targeted by the vaccine sequences, as confirmed by PCR; it will be completed in March 2024. NCT05606965 is an ongoing phase 2 study is comparing mRNA-1010 against a broader spectrum of comparator vaccines, in two small populations with 18–50 years or 65–80 years of age and using a broader set of outcomes; it is expected to be completed by September 2023. Regarding the competing mRNA vaccine qIRV (22/23), a phase 3 study has recently completed enrollment of 36,454 subjects and will be completed by August 2023; the focus was on a single dose and shot of mRNA vaccine used against licensed vaccines, and on the two subpopulations aged 18–64 years and 65 or more. The main outcome is a laboratory-confirmed influenza-like illness and antibody titration, plus safety, and comparisons among vaccines efficacy will be carried out. It remains to be determined if and which of the two competitors will show a better efficacy than that of traditional vaccines.

Respiratory syncytial virus is a severe health threat for immunocompromised patients and for fragile persons, especially the elderly and infants below the age of 5 years; it is estimated to cause around 100 thousand deaths per year just among infants [146]. It is not currently targeted by specific antivirals or by vaccines, although traditional vaccine candidates have been developed with different approaches; however, all resulted in being inefficacious and/or in causing intolerable adverse reactions in many treated subjects, including a worsening of symptoms of later infections [147]. Therefore, mRNA-1345 could be the first effective vaccine for the respiratory syncytial virus. NCT05330975 is a phase 3 study administering one fixed dose of mRNA-1345 versus placebo, together with an updated mRNA vaccine against SARS-CoV-2 or together with a quadrivalent anti-influenza traditional vaccine. It recruited 3354 adults over 50 years of age and is planned to be completed in May 2023. Its outcomes include seroprevalence and antibody titers for all targeted viruses, and safety measures. NCT05127434 is an ongoing large study composed of a phase 2 (with 2000 subjects) and a phase 3 part (with 35,000 subjects). It is focused on subjects aged 60 or more and it will collect outcomes of safety, alongside the occurrence of a PCR-confirmed symptomatic first infection by respiratory syncytial virus. It is expected to end by November 2024.

Varicella zoster virus is a serious health threat due to the importance of its consequences, especially considering post-herpetic neuropathy. It is currently targeted by a live attenuated vaccine and by a recombinant vaccine; their efficacy is reported to be 84% at best, with two administrations of the recombinant one in immunocompetent subjects, but it can be absent in immunocompromised individuals using the live attenuated one [148]. Moreover, the vaccine effectiveness in preventing infection symptoms is reported to be generally lower [149] and the prevention of post-herpetic neuralgia remains an unmet medical need. Therefore, three mRNA vaccine candidates are being developed together in the ongoing phase 1/2 trial NCT05703607. It is recruiting 900 subjects of 50 to 69 years of age, who will be randomized to receive three different mRNAs that will be used either with immediate dilution from powder or by thawing a frozen vial. Vaccination will require two injections and the comparator treatment will be the licensed recombinant vaccine. There will be an extensive collection of safety main outcomes, plus secondary outcomes of efficacy, and the study is planned to end by 2030.

Zika virus has the ability to cross the placental barrier and infect the fetus in around 25% pregnancies from infected mothers, causing in certain cases fetal abnormalities, including central nervous system defects in around 6% of cases, small for gestational age in around 4% of cases, and premature birth in around 7% of cases [150]. There are currently several vaccine candidates in clinical development, both traditional and DNA or RNA based, but none is yet approved. Among these, is mRNA-1893, which has been tested in a phase 2 study on 809 subjects, which will end by April 2024. Treatment consists of two administrations of low or high dose mRNA-1893, versus placebo, given to healthy adults not willing to become pregnant. The main outcomes include safety measures, antibody titers, and seroconversion rates.

Papilloma virus is a global health concern, responsible for causing around 90% of cervical and anal cancers, and 60–70% of cancers of penis, vagina, and oropharynx. Traditional vaccines currently available involve two or three administrations and compliance to vaccination remains very low worldwide, also considering economically wealthy countries; vaccine efficacy is relatively high in immunocompetent young subjects, for instance it is reported to produce risk rate ratios of around 0.10–0.17 versus control, regarding the occurrence of different kinds of neoplasms in males [151]. Besides the possibility of adverse reactions, the main issue with papilloma virus vaccination is therefore accessibility and compliance to repeated administration. The candidate mRNA vaccine BNT113 is undergoing the phase 2 study NCT04534205, which aims to recruit 285 subjects. It will be used in combination with pembrolizumab, for the treatment of unresectable or metastatic head and neck squamous cell carcinoma expressing PD-L1, against pembrolizumab alone. Of interest, BNT113 will be administered only once. Outcomes will be assessed up to 4 years after injection and include a broad spectrum of oncological efficacy markers and safety measures. The study is foreseen to end in 2028.

Regarding non-virus-related cancer, neoantigens are the target of both peptide and mRNA vaccines. Study NCT05456165 was testing a patient-specific self-amplifying mRNA against neoantigens from colorectal cancer patients; the trial has been terminated early due to reprioritization and only one patient was recruited. For melanoma, the two mRNA-4157 and BNT111 are being tested in phase 2 on resected and unresectable disease; being targeted against different disease stages, they therefore may not become competitors. Study NCT03897881 is conducted on mRNA-4157, which encodes up to 34 cancer neoantigens, used as adjuvant treatment in combination with pembrolizumab and against pembrolizumab alone, in 157 patients with resected high-risk melanoma. Treatments comprise 9 administrations of mRNA-4157 and up to 18 administrations of pembrolizumab, each one every 21 days. The main outcome is recurrence-free survival, with several secondary outcomes of oncological interest, and the trial is foreseen to end by September 2024. Study NCT04526899 is recruiting 180 patients with anti-PD-1 refractory or unresectable advanced melanoma, treating them with BNT111 and/or cemiplimab in single administration. BNT111 encodes four neoantigens commonly expressed in melanoma cells. The main outcome is response rate, up to 24 months from injection, followed by many oncological secondary outcomes; the study will be completed by June 2025.

## 8. Challenges and Remarks

The main advantage of RNA-based therapeutics, including mRNA vaccines, is their ability to specifically target disease-causing genes/proteins while leaving healthy cells unaffected [4,5]. This makes them potentially safer and more effective than traditional drugs. RNA-based therapeutics may act on targets that are otherwise undruggable for other drugs and can be rapidly developed with relative lower costs in comparison to those of small molecules or recombinant proteins. In addition, the plasticity of RNA-based construct and its quick effect in biological systems make it useful for personalized treatments or to adapt to an evolving pathogen/disease [4,5]. However, while RNA-based therapeutics hold great promise in a wide range of diseases, there are also several challenges associated with the development and use of these drugs. Some of the main issues include:

(i) Stability: RNA molecules are inherently unstable and can be degraded quickly by enzymes in the body;

(ii) Delivery: RNA molecules are large and negatively charged, which makes it difficult for them to penetrate cells and tissues. In addition, although a huge variety of delivery systems have been employed, none of them is cell specific. Targeting antigens to specific cells, APCs for instance, might be crucial to enhance vaccination efficacy while decreasing adverse effects;

(iii) Immunity: it is unclear which aspects of innate immunity are essential for promoting protective immune responses and which are not needed. A detailed mechanical understanding of how the innate immune systems modulate adaptive immunity in mRNA vaccine responses is also missing;

(iv) Immunogenicity: some types of RNA molecules can trigger an immune response in the body, which can lead to side effects or reduce the effectiveness of the drug;

(v) Off-target effects: RNA-based drugs can potentially affect genes or proteins other than their intended target, leading to unintended side effects;

(vi) Persistence of mRNA-LNP in the blood and presence in the breast milk [152]: few studies have been focused on these peculiar aspects that can be either positive or negative. Complex human pharmacokinetics studies are necessary to unravel the tropism of mRNA vaccine particles;

(vii) Manufacturing: RNA-based drugs require specialized manufacturing processes, which can be expensive and time consuming;

(viii) Intellectual property: developing RNA-based drugs requires significant investment, but the ability to protect intellectual property for these drugs can be challenging due to the complexity and rapid evolution of the technology.

### 8.1. Lesson from Pfizer-BioNTech Comirnaty and the Moderna Spikevax

Despite the proved success of SARS-CoV-2 mRNA vaccines, further challenges regarding this type of vaccine remain to be addressed. For instance, the above mentioned self-adjuvating properties of the current mRNA/LNP vaccines need to be considered as a double-edged sword. Indeed, the recognition by the innate immune system can have detrimental consequences on vaccine efficacy and therefore on disease treatment, thus limiting the potential of mRNA vaccines. On the other hand, the inflammation triggered by the LNP platform may have beneficial effects on vaccine efficacy, if appropriately tuned [153]. The role of the adjuvant properties of mRNA vaccines is still debated and balancing an efficient antigen production with adjuvant effects and putative side effects is still an open challenge that needs to be addressed. Co-encapsulation of other adjuvants or agonists has been proposed and is still under investigation [23,73].

The rapid and continuous mutation of the vaccine-selected antigens has been a strong limitation for traditional vaccines in both infectious and cancer-related diseases. Although mRNA vaccines can theoretically be easily adapted, the immune escape of mutated antigens and the vaccine-related selective pressure exerted on these remain the most challenging issues [154,155]. Indeed, the waning vaccine-elicited protection against the new SARS-CoV-2 variants observed during the recent pandemic was, and still is, a lesson to learn. Moreover, the duration of the vaccine-elicited protection is still debated, and it greatly varies according to the pathogen. So far, the consensus on the protection from SARS-CoV-2-related hospitalization or death falls in the order of magnitude of months, probably less than six [84,156]. Although not directly correlated, plasmatic Ab titer follows this same timing. Recall booster doses are very effective when considering the plasmatic Ab titer, but not as much when considering the duration of the protection over 3–6 months [156]. Also, the dynamics of the immune system in response to SARS-CoV-2 mRNA vaccine are still largely undefined in subjects bearing underlying conditions, namely patients diagnosed with malignancies, chronic infections (HIV, HCV, etc.), neurological disorders, immunological disorders, or even in the particular setting of pregnancy. Understanding the vaccine-elicited immune reactions would possibly be of paramount importance for clinical management of these cohorts and for defining appropriate public health guidelines for vulnerable populations, including dedicated fine-tuned specific vaccinal plans [80,157,158,159,160].

### 8.2. Perspectives

**Figure 3:**
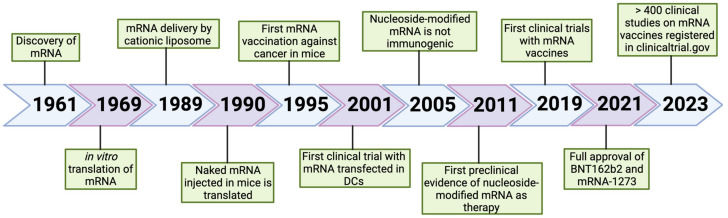
Timeline of the key advances in the pharmacological development of mRNA-based therapies. The discovery of RNA dates back to 1961 [161]. Key steps in mRNA-based therapy are the possibility to translate the exogenous mRNA in vitro and the discovery of liposomes as delivery systems [162,163] The first in vivo experiments were performed in murine models [164,165], followed by a clinical trial with ex vivo transfected DCs [166]. Another fundamental advance towards the use of mRNA in a clinical setting was the finding that nucleoside-modified mRNA is not immunogenic [71]. This paved the way to the first preclinical study with this type of mRNA as therapeutic drug [167]. From 2019 to the present several clinical trials have been registered, with the first two vaccines fully approved in 2021 [75,78]. Created with Biorender.com.

Since 1961 when it was discovered [161], mRNA has been the focus of consistent basic and applied research for various diseases (Figure 3). mRNA vaccines are a great advancement in the field of vaccination also showing significant superiority over other types of vaccines. Currently, a great deal of research focuses on varied applications and a series of pharmacological investigations and clinical trials are ongoing. Undoubtedly, opportunities and challenges coexist in the therapeutic potential of mRNA vaccines but there are also a large number of questions requiring clarification. In this respect, thorough research is still necessary to fill the scientific and regulatory gaps. Built on the highly fueled interest and potential, we have full confidence that these developments will provide many solutions for the prevention and treatment of a variety of infectious diseases and cancers.

## Figures and Tables

**Table 1:** Number of clinical trials involving mRNA vaccination, grouped by target disease (source: clinicaltrials.gov) (accessed on 1 April 2023).

	Disease	Completed	Active, Not Recruiting	Recruiting	Not yet Recruiting	Terminated/Unknown	Total
Cancer	Advanced solid		1	2	1		4
	AML	1					1
	Breast		1				1
	EBV related			2			2
	Gastrointestinal			1	1	2	4
	Glioblastoma	3	1	1	1		6
	Melanoma	2	2	1	1		6
	NSCLC	2					2
	Prostate	1					1
	other	1			2	2	5
Viral infections	Cytomegalovirus	2	1	3	1		7
	Epstein–Barr			1			1
	Hepatitis B	1					1
	Herpes simplex 2			1			1
	Varicella zoster			2			2
	HIV	1	3			1	5
	Influenza	2	4	8			14
	Metapneumovirus	2					2
	Nipah			1			1
	Papilloma			2			2
	Rabies	2					2
	Respiratory Syncytial		2	4			6
	Zika	2	1				3
	Influenza + SARS-CoV-2			1			1
	Plasmodium falciparum			1			1
	Mycobacterium tuberculosis	1			2		3
							
	Total excluding SARS-CoV-2	23	16	31	9	5	84
	Viral infections by SARS-CoV-2	72	87	116	35	22	332
							
	Total	95	103	147	44	27	416

**Table 2:** Prevalence of active studies on mRNA vaccines among studies, grouped by target disease (source: clinicaltrial.gov) (accessed on 1 April 2023).

	Disease	Active st. mRNA /Active Studies	Active st. mRNA/Vaccine Studies	Active st. mRNA/Active Vaccine st.
Cancer	Advanced solid	0.1%	10.5%	14.8%
	EBV related	9.1%	50%	100%
	Gastrointestinal	<0.1%	1.3%	3.6%
	Glioblastoma	0.5%	3.4%	6.8%
	Melanoma	0.4%	1.7%	10.8%
Viral infections	Cytomegalovirus	5.5%	12.5%	29.4%
	Epstein–Barr	0.8%	7.1%	16.7%
	Herpes simplex 2	4.0%	8.3%	2.6%
	Varicella zoster	2.2%	1.0%	4.4%
	HIV	0.2%	0.6%	3.3%
	Influenza	4.0%	0.8%	8.4%
	Nipah	50.0%	33.3%	50%
	Papilloma	1.6%	1.6%	5%
	Respiratory Syncytial	7.7%	4.6%	14.0%
	Zika	6.7%	3.7%	33.3%
	Influenza + SARS-CoV-2	1.4%	5.9%	8.3%
	Plasmodium falciparum	1.3%	0.6%	3.2%
	Mycobacterium tuberculosis	0.7%	1.4%	6.5%
	Viral infections by SARS-CoV-2	7.0%	17.7%	26.1%

**Table 3:** Randomized controlled trials in phase 2 or 3 conducted on diseases other than SARS-CoV-2 (source: clinicaltrial.gov) (accessed on 1 April 2023).

	Disease	Phase 3 RCTs		Phase 2 RCTs	
Cancer	Gastrointestinal	0		1	NCT05456165
	Melanoma	0		2	NCT03897881; NCT04526899
Viral infections	Cytomegalovirus	1	NCT05085366	2	NCT04232280; NCT05683457
	Varicella zoster	0		1	NCT05703607
	Influenza	3	NCT05415462; NCT05540522; NCT05566639	1	NCT05606965
	Papilloma	0		1	NCT04534205
	Respiratory Syncytial	2	NCT05127434; NCT05330975 (phase 2/3)	0	
	Zika	0		1	NCT04917861

## Data Availability

All published data is contained within the article.
